# The epigenetic basis for the impaired ability of adult murine retinal pigment epithelium cells to regenerate retinal tissue

**DOI:** 10.1038/s41598-019-40262-w

**Published:** 2019-03-07

**Authors:** Galina Dvoriantchikova, Rajeev J. Seemungal, Dmitry Ivanov

**Affiliations:** Bascom Palmer Eye Institute, Department of Ophthalmology, University of Miami Miller School of Medicine, Miami, FL 33136 USA

## Abstract

The epigenetic plasticity of amphibian retinal pigment epithelium (RPE) allows them to regenerate the entire retina, a trait known to be absent in mammals. In this study, we investigated the epigenetic plasticity of adult murine RPE to identify possible mechanisms that prevent mammalian RPE from regenerating retinal tissue. RPE were analyzed using microarray, ChIP-seq, and whole-genome bisulfite sequencing approaches. We found that the majority of key genes required for progenitor phenotypes were in a permissive chromatin state and unmethylated in RPE. We observed that the majority of non-photoreceptor genes had promoters in a repressive chromatin state, but these promoters were in unmethylated or low-methylated regions. Meanwhile, the majority of promoters for photoreceptor genes were found in a permissive chromatin state, but were highly-methylated. Methylome states of photoreceptor-related genes in adult RPE and embryonic retina (which mostly contain progenitors) were very similar. However, promoters of these genes were demethylated and activated during retinal development. Our data suggest that, epigenetically, adult murine RPE cells are a progenitor-like cell type. Most likely two mechanisms prevent adult RPE from reprogramming and differentiating into retinal neurons: 1) repressive chromatin in the promoter regions of non-photoreceptor retinal neuron genes; 2) highly-methylated promoters of photoreceptor-related genes.

## Introduction

Since nearly 80 percent of sensory information is collected by means of sight, vision loss resulting from traumatic injuries or diseases has significant economic and moral impacts on all levels of society^1–3^. Current treatment paradigms, while diverse in their pharmacological targets, are all essentially predicated on slowing the rate of degenerative change—a “cutting your losses” approach. Meanwhile, new approaches for restoring sight, including transplants of stem cells or their differentiated derivatives, and gene therapies, have already demonstrated promising results, but all depend on invasive ophthalmologic surgical techniques^4–8^. An ideal reparative strategy for the retina would be for it to heal itself – an ability employed by many species, but is known to be absent in mammals.

Adult teleost fish, such as zebrafish, and amphibians (*X*. *laevis*, *Cynops pyrrhogaster*) have an outstanding ability to regenerate damaged retina and restore lost sight^9–14^. The retinal regeneration in these species relies on two different cell types: Müller glia (teleost fish) and retinal pigment epithelium (RPE, amphibians)^9–14^. Adult Müller glia and RPE undergo a reprogramming process allowing them to generate progenitors for retinal regeneration after injury in teleost fish and amphibians, however the reason why this ability is absent in mammals is not yet understood^9–14^. RPE and Müller glia are derivatives of optic vesicle progenitors (OVPs)^15–20^. It was shown that the OVPs, in the absence of signals from the surface ectoderm, differentiate into RPE^15–20^. However, when the surface ectoderm comes into close contact with the optic vesicle, it promotes retinal phenotypes in these progenitors, which differentiate into six types of retinal neurons and one glial type (Müller glia) during retinal development^15–22^. Thus, RPE may be epigenetically very close to OVPs and may have the ability to regenerate the entire retina, but this is not found in mammals. In the past 10 years, significant progress has been made in the scientific understanding of the adult Müller glia’s capacity to regenerate injured retina on many levels, including epigenetic^11,23–25^. Meanwhile, the ability of adult RPE to regenerate damaged retina has been explored less. In particular, there have been no in-depth epigenetic studies of adult RPE. In this study, we evaluated the epigenetic plasticity of adult murine RPE to identify possible mechanisms that prevent mammalian RPE from regenerating the entire retina.

## Results

### Global epigenetic profile of adult murine RPE

**Figure 1:**
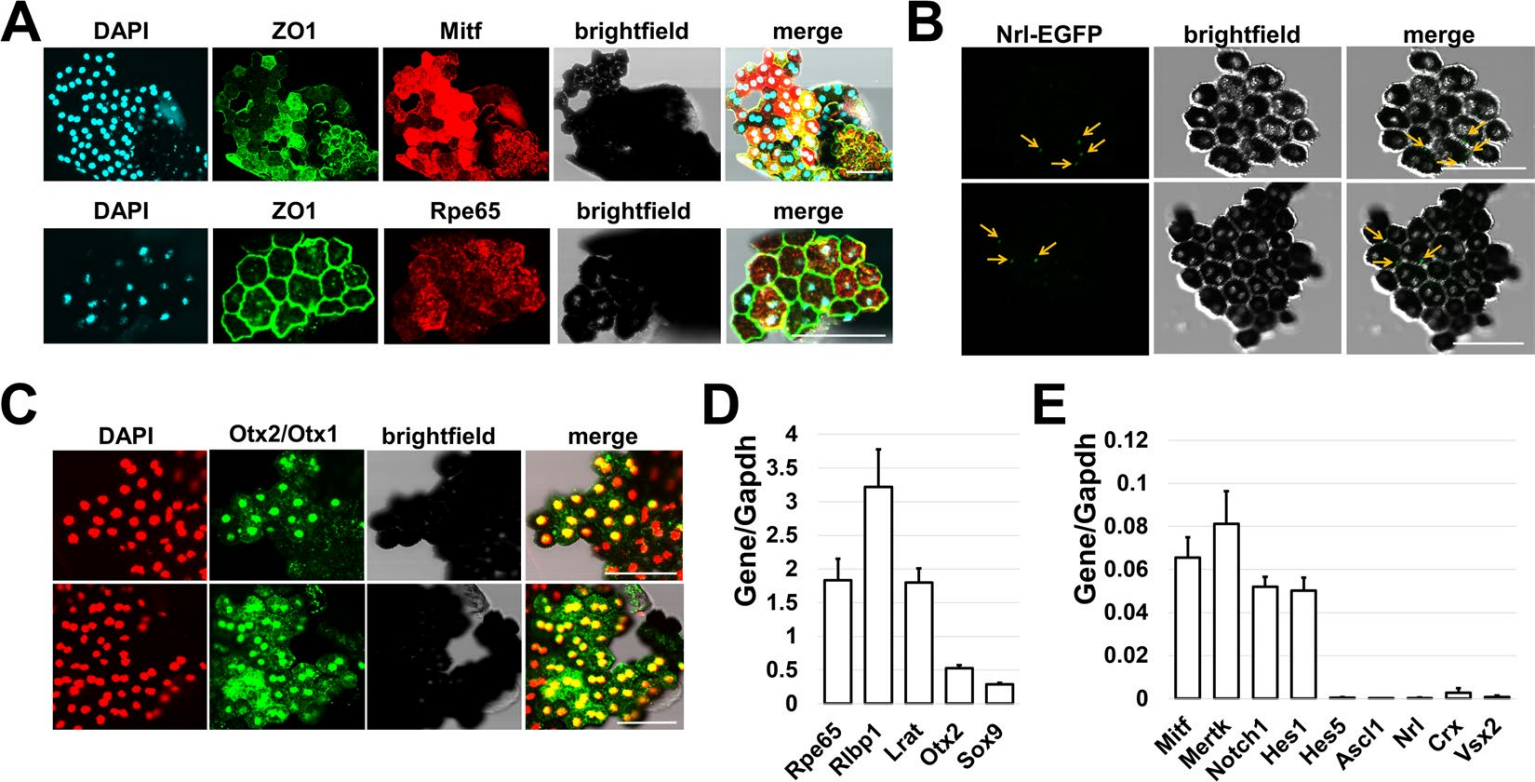
The detaching of uninjured RPE sheets from murine eyecups is an effective approach for isolation of highly pure RPE cells. (**A**) Immunohistochemistry showed high protein levels of RPE markers, Mitf and Rpe65, in isolated cell sheets and that pigmented cells form tight junctions (ZO1 marker) with each other. 4′,6-Diamidino-2-phenylindole (DAPI) was used to label DNA, and thus allowed visualization of the cell nucleus. Bar is 50 μm. (**B**) RPE sheets isolated from Nrl-EGFP animals (these animals have EGFP labeled rod photoreceptors) show no contamination with rod photoreceptors. Bar is 50 μm. (**C**) Antibodies against Otx2 and Otx1 were used to identify RPE in cell sheets. Since Otx2 and Otx1 are transcription factors, they were localized in the cellular nucleus (DAPI as a marker). Bar is 50 μm. (**D**,**E**) Expression of RPE and retinal markers in RPE sheets was evaluated by qRT-PCR. For each gene, the results are expressed as a fold-change of the corresponding value for Gapdh (housekeeping gene) ±SE of the mean (n = 6).

To study the epigenetic profile of adult RPE, we used cells from 2.5–3 month old mice. We employed the protocol developed by Dr. Fernandez-Godino *et al*. published in Nature Protocols to isolate RPE^26^. The collection of uninjured RPE sheets detached from eyecups takes less than 3 hours. To confirm the purity, we tested the expression of RPE markers and compared it to the expression of retinal cell type markers, including markers of photoreceptors (*Crx*, *Nrl*) that are in close contact with RPE. The isolated RPE sheets contained the squamous and polygonal pigmented cells (Fig. 1A–C). The integrity of the isolated RPE sheets was indicated by the expression of the tight-junction protein ZO1. (Fig. 1A). Immunostaining with an antibody against Mitf, Rpe65, and Otx2 showed that isolated cells expressed all of the aforementioned RPE markers (Fig. 1A,C). To detect contamination with rod photoreceptors, which are closest in proximity to RPE, we used RPE sheets isolated from genetically modified Nrl-EGFP animals. We found small regions of fluorescence (diametrical size of 1–2 µm) that might be the fragments of photoreceptor outer segments (Fig. 1B), but not fully intact photoreceptors of significantly larger size. Using quantitative RT-PCR, we also compared the levels of gene expression for RPE markers and non-markers in isolated cell sheets. We found significantly high expressions of genes related to the cellular functions of RPE (*Rpe65*, *Rlbp1*, *Lrat Otx2*, *Sox9*, *Mitf*, *Mertk*, *Notch1*, *Hes1*). Meanwhile, the expression of all non-RPE markers including *Vsx2* (Chx10; a retinal progenitor cell (RPC) and bipolar cell marker – an inhibitor of the RPE phenotype), *Crx* (photoreceptor marker), *Nrl* (rod photoreceptor marker), *Hes5* (Müller glia marker), and *Ascl1* (RPC marker, found in almost all retinal phenotypes except RGCs) was negligible (Fig. 1D,E). Overall, the approach used in our study proved to be an effective technique for the isolation of high purity RPE cells.

**Figure 2:**
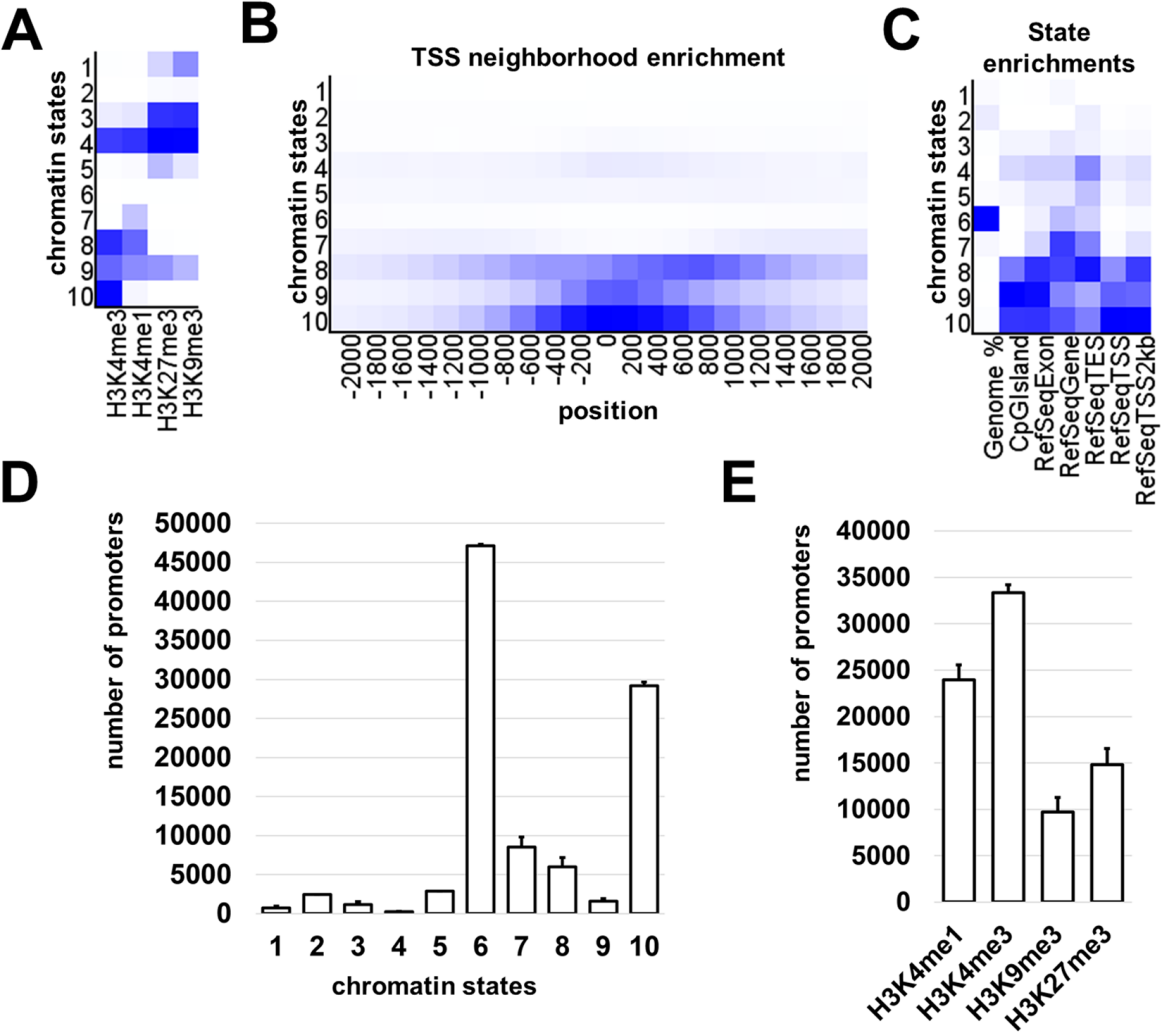
ChIP-seq analysis revealed 10 chromatin states in adult RPE. (**A**) Heat map of the chromatin states, which were identified by the chromHMM software package. The darker blue color labels abundant ChIP-seq marks in the chromatin state. (**B**) The fold enrichment for each chromatin state at fixed positions (from −2000 bp in the promoter area up to 2000 bp in the first exon and intron) relative to the transcription start site (TSS) indicates highly modified histone (mostly active H3K4me3 marks) accumulation around the TSS in RPE genome regions in chromatin states 8, 9, and 10. (**C**) Heat map for chromatin state functional enrichments shows the genome, CpG islands, exons, genes, transcript end sites (TES), TSS, and 2000 base pair intervals around the TSS represented by each state. (**D**) The columns indicate the number of promoters located in each chromatin state. (**E**) The ChIP-seq data was annotated and presented as the number of promoters containing H3K4me1, H3K4me3, H3K9me3, and H3K27me3 marks.

To comprehensively characterize the epigenetic states of the RPE isolated from adult animals, we analyzed these cells on different levels: (1) expression level using microarrays; (2) genome-wide histone modifications using ChIP-seq technology; (3) DNA methylation using the whole-genome bisulfite sequencing (WGBS) approach. In order to identify genes expressed in adult RPE we used Mouse Exonic Evidence Based Oligonucleotide (MEEBO) microarrays, which included 38,083 genes and transcripts. We processed individual samples that contained 150,000–200,000 cells each. Three independent biological replicates were obtained for comprehensive gene expression profiling of the adult RPE. The results of our microarray analysis indicated that a total of 8410 genes passed the quality control criteria and statistical significance tests to identify genes expressed in adult murine RPE (Supplementary Data S1). In our analysis, we detected high expressions of RPE cell markers (Supplementary Data S1). Using the KEGG PATHWAY database, we observed that a significant number of genes from the cell cycle, Hippo, WNT, TGFβ, NOTCH, Insulin, MAPK, and Glioma signaling pathways were highly expressed in adult RPE (Supplementary Data S1). To study genome-wide histone modifications in adult RPE we chose four histone marks: H3K4me3, H3K4me1, H3K27me3, and H3K9me3; each of which, or certain combinations, characterize the chromatin epigenetic state: the active/open (permissive) chromatin (H3K4me3 only or in combination with H3K4me1), the bivalent/poised state (H3K27me3 and H3K4me3), the temporarily inactive (repressive) Polycomb state (marked by H3K27me3), and the permanently inactive (repressive) state (H3K9me3 only or in combination with H3K27me3). Two pellets, from independent biological replicates, of fixed RPE samples containing 150,000–200,000 cells were used. The chromatin was extracted and sheared from the cells of each sample. Then 4 ChIP assays (H3K4me1, H3K4me3, H3K9me3, and H3K27me3) were performed on each chromatin preparation, followed by sequencing, alignment, and peak calling procedures for studied histone modifications (Supplementary Data S2). To identify the chromatin states, we used computational, multivariate Hidden Markov Models (chromHMM) with all of our ChIP-seq data^27^. This allowed us to annotate the epigenetic chromatin states across the entire adult RPE mouse genome (Fig. 2 and Supplementary Data S3). Based on this computational approach, we chose 10 chromHMM chromatin states for examination (Fig. 2A). Chromatin states 1 and 3 represent the H3K9me3 and H3K27me3 markers – permanently inactive (repressive) chromatin. States 2 and 6 were empty (open) chromatin. State 4 was different from other states, because it was marked by all histone modifications tested in the study. Analysis of gene promoters related to state 4, as well as RPE gene expression, suggested that state 4 might define active genes located in inactive regions. Chromatin state 5 represents the polycomb-repressed chromatin (H3K27me3) marker. State 7 could be predominantly enhancers, since H3K4me1 is mostly an enhancer mark^28^. States 8 and 10 had epigenetic marks of active genes (permissive states) and state 9 may designate bivalent/poised promoter regions (Fig. 2). The numbers of gene promoters located in regions with corresponding chromatin states and individual histone marks are shown in Fig. 2D,E. The data indicate that the majority of RPE promoters are in open (no tested histone marks) or active chromatin, which is characteristic of epigenetically mobile stem cells and progenitors^29–31^.

**Figure 3:**
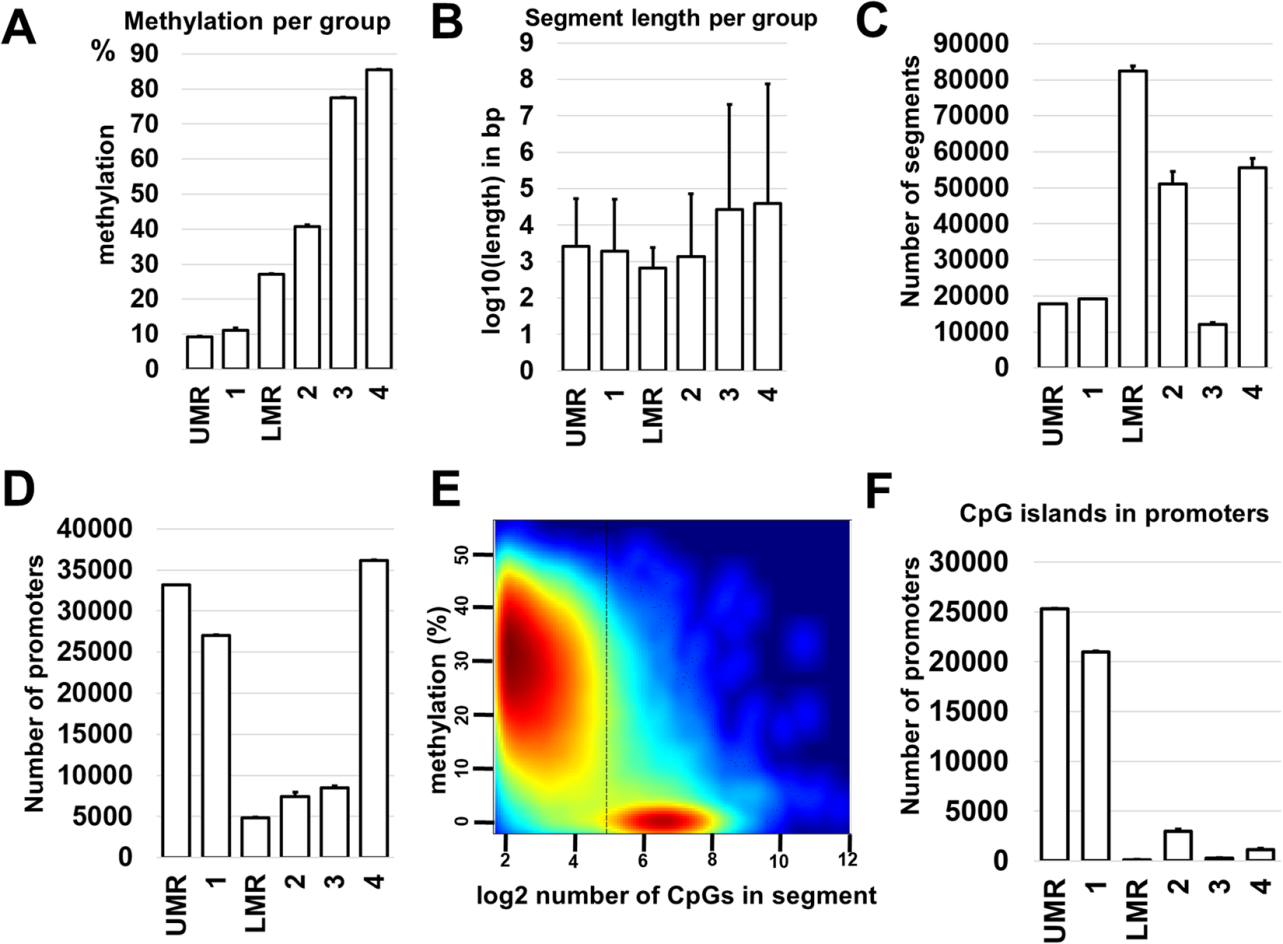
Hidden Markov Model (HMM)-based methods (MethylSeekR) and change-point-based methods (methylKit) are robust and comparable approaches for segmentation of the RPE methylome. (**A**) Average methylation levels were determined for each segmentation class and methylation region. (**B**) Average segment and region lengths (log10 transformed base pairs; bp) were calculated. (**C**) The average number of segments vary between segmentation classes and methylation regions. (**D**) The majority of promoters belongs to unmethylated (UMR and segmentation class 1) or highly-methylated (segmentation class 4) areas of the RPE genome. (**E**) MethylSeekR allowed us to separate CpG-rich, unmethylated UMRs, and CpG-poor LMRs. The figure generated by the MethylSeekR package shows the number of CpGs (hypomethylated regions) versus its average methylation level. (**F**) The CpG islands identified in RPE promoters mostly belong to unmethylated regions (UMR and segmentation class 1).

Our study of DNA methylation involved two WGBS libraries prepared using genomic DNA from two independent RPE samples containing 150,000–200,000 cells. The WGBS libraries were sequenced, and bisulfite sequence reads were aligned using the Bismark software package. Global methylation levels in adult mouse RPE were then measured and the percentages of methylated CpG, CHG, and CHH contexts (where H is A, C or T) were found to be 79% for CpG, 0.6% for CHG, and 0.6% for CHH. Since most of the adult RPE genomic DNA methylation was restricted to symmetrical CpG sequences, and the role of CpG methylation in mammalian genomes is very well studied, while the role of CHG and CHH methylation is not yet understood, we focused our analysis on CpG methylation in the RPE genome. To analyze methylome segmentation within RPE genomic DNA, we used computational approaches. We first used the R package “MethylSeekR”, a computational tool to accurately identify unmethylated regions (UMRs), low-methylated regions (LMRs), and partially methylated domains (PMDs) from bisulfite-sequencing data (Supplementary Data S4)^32^. The methylome feature of PMDs was found in some cells and tissues^33,34^. However, the majority of cell and tissue methylomes do not possess this feature^33,34^. Our data indicates that PMDs are absent in the methylome of adult murine RPE (Supplementary Data S5). Next, we segmented the RPE methylome into four distinct features (segmentation classes) using the methylKit package, which identifies segments based on only the average DNA methylation level (Supplementary Data S4)^35^. It was previously shown that there exists a high-concordance between the MethylSeekR and methylKit computational approaches (segment classes 1 and UMRs, as well as segment classes 2 and LMRs largely share the same regions and have similar segment lengths as well as methylation levels)^36^. Meanwhile, segment classes 3 and 4 correspond to highly-methylated regions^36^. These results are consistent with our data (Fig. 3A–C, Supplementary Data S4). Annotation of identified segments and regions was carried out with the R-based Bioconductor package: “Annotatr”^37^. We found that the gene promoters were located in either highly-methylated or unmethylated regions (Fig. 3D, Supplementary Data S4). CpG islands in the promoter area of the genes were mostly unmethylated (82% of CpG islands had an average methylation lower than 10%) (Fig. 3E,F, Supplementary Data S4). It should be noted that many studied promoters do not contain known CpG islands (Supplementary Data S4).

### Adult mammalian RPE cells are epigenetically close to progenitor-like cell types and predisposed to an epithelial-mesenchymal transition (EMT)

**Figure 4:**
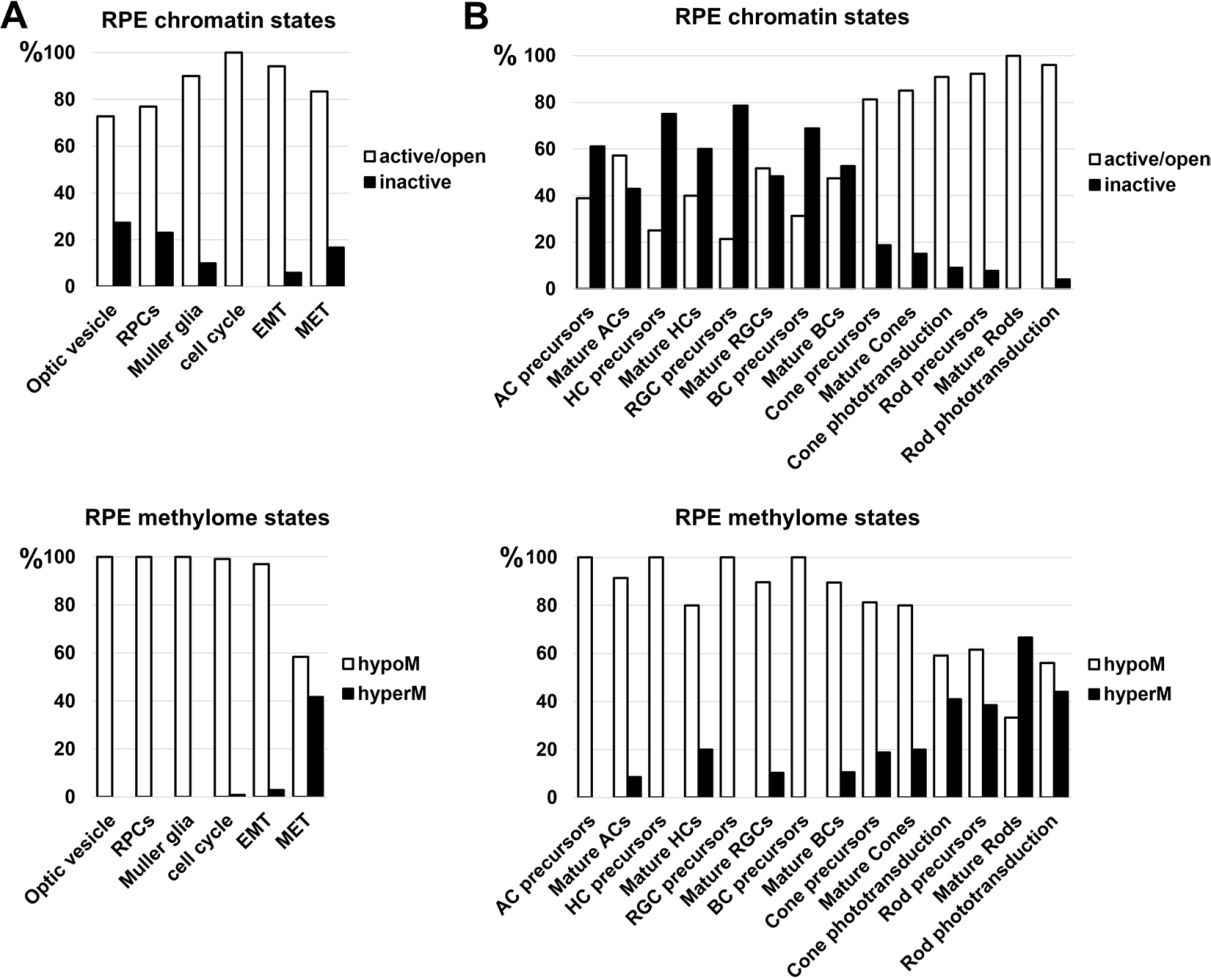
The chromatin and methylome states of key genes involved in development and function were evaluated in the genome of adult RPE. (**A**) The chromatin states and methylome states of key genes required for progenitor-like phenotypes (optic vesicle progenitors, RPCs, Müller glia), the cell cycle, EMT, and MET are relatively similar in adult RPE. (**B**) The non-photoreceptor retinal neuron phenotypes have the majority of gene promoters in an inactive (repressive) chromatin state, but these promoters belong to unmethylated or low-methylated regions. Meanwhile, promoters of photoreceptor-related genes are in an active/open (permissive) chromatin state, but are highly-methylated. (% - percent of genes in the respective states; hypoM - hypomethylated genes; hyperM - hypermethylated).

Using our global epigenetic profile of adult RPE, we compared our data with lists of key genes required for optic vesicle progenitor (OVP), retinal progenitor cell (RPC), and Müller glia development. The key genes required for optic vesicle development were collected from peer-reviewed articles (PubMed – NCBI). The lists of genes that regulate RPC and Müller glia development were obtained from LifeMap Discovery and The Stem Cell Research Database. Results of our analysis indicate that 73% of genes required for optic vesicle development were in an active/open (permissive) chromatin state, while 27% were in an inactive (repressive) chromatin state (Fig. 4A, Supplementary Data S6). However, all genes in an inactive (repressive) state were in a temporarily inactive Polycomb state (marked by H3K27me3), which may be activated in the presence of pioneer transcription factors (PTFs) (Fig. 4A, Supplementary Data S6). The majority of genes required for RPC and Müller glia development were in open or active (permissive) states (77% and 90% respectively, Fig. 4A, Supplementary Data S6). Meanwhile, our analysis of the methylation states for promoters of OVP, RPC, and Müller glia genes revealed that they were in unmethylated or low-methylated regions of the RPE genome (Fig. 4A, Supplementary Data S7). Thus, adult RPE are epigenetically very close to progenitor-like cell phenotypes.

Proliferative vitreoretinopathy (PVR) is a result of diseases in which RPE cells proliferate and undergo an epithelium-to-mesenchyme transition (EMT), resulting in fibroblastic mesenchymal progeny that proliferate on the surface of the retina, facilitating retinal damage^38–40^. To explain this behavior of RPE under pathological conditions, we evaluated epigenetic states of genes required for the cell cycle, EMT, and mesenchyme-to-epithelium transition (MET). The lists of genes required for EMT, MET, and the cell cycle were obtained from Gene Ontology (GO) Consortium, KEGG database, and peer-reviewed articles (PubMed – NCBI). We found that almost all EMT, MET, and cell cycle related genes were in an active/open (permissive) chromatin state (94%, 83% and 100% respectively, Fig. 4A, Supplementary Data S6). Promoters of EMT and cell cycle related genes were mostly unmethylated or low-methylated (Fig. 4A, Supplementary Data S7). Meanwhile, 42% of promoters of MET related genes were highly-methylated (Fig. 4A, Supplementary Data S7). Thus, our data indicate that adult mammalian RPE cells are predisposed to PVR.

### The majority of non-photoreceptor genes had promoters in a repressive chromatin state in adult RPE

**Figure 5:**
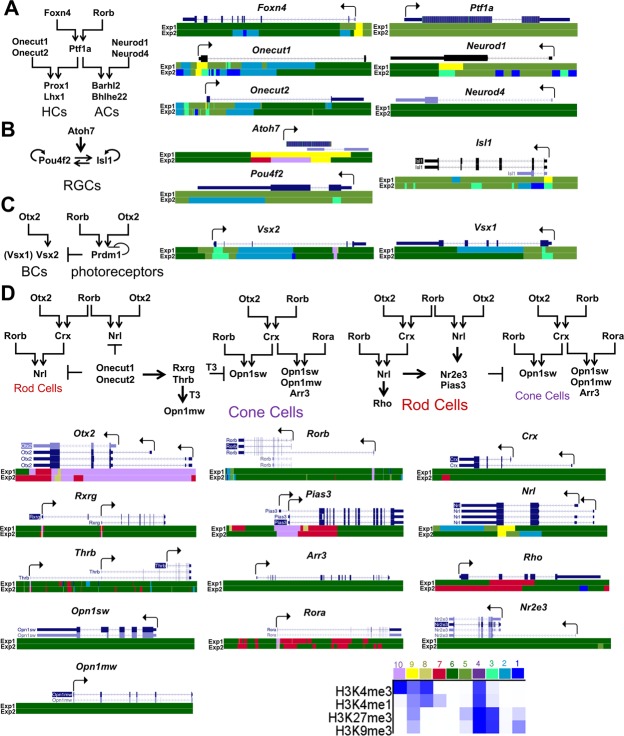
HHM states were identified in adult RPE for key genes required for horizontal and amacrine cell (**A**), RGC (**B**), bipolar cell (**C**) and photoreceptor (**D**) differentiation. Promoters of many genes that regulate all retinal neuron (except photoreceptor) development belong to regions in chromatin states 3, 5 (majority of genes), and 9. The chromatin states 5 and, partially, 9 (depending on the levels of H3K4me3 and H3K27me3) are temporally inactive. The chromatin state 3, marked by both H3K9me3 and H3K27me3, is related to a permanently inactive (repressive) chromatin state. Promoters of the majority of genes that regulate cone and rod photoreceptor development belong to regions in chromatin states 6, 7, 8, and 10, allowing gene expression.

Using our ChIP-seq data, we evaluated the epigenetic ability of adult RPE to differentiate into retinal neurons. We also evaluated genes required for rod and cone phototransduction. The lists of genes that regulate the development of all retinal cell types were obtained from LifeMap Discovery and The Stem Cell Research Database. The list of genes required for phototransduction was extracted from the KEGG database and RGD database (https://rgd.mcw.edu/wg/). Analysis of chromatin states of key genes required for development of retinal neurons revealed that *Foxn4* and *Ptf1a*, key transcription factors that regulate the phenotype of retinal inhibitory neurons (horizontal and amacrine cells), were in an inactive (repressive) chromatin state (Fig. 5A, Supplementary Data S6). Expression of both genes was not detected in RPE (absence of *Foxn4* expression was shown by qRT-PCR). The majority of genes required for formation of precursors and mature horizontal and amacrine cells were in an inactive (repressive) chromatin state (61% of amacrine cell precursors, 75% of horizontal cell precursors; 43% of genes required for mature amacrine cell development; 60% of mature horizontal cells) (Fig. 4B, Supplementary Data S6). Thus, the ability of adult mammalian RPE to differentiate into inhibitory types of retinal neurons may be very low, since many key factors should be epigenetically activated. We also analyzed the chromatin states of genes regulating excitatory types of retinal neurons (retinal ganglion cells (RGCs), bipolar cells, cone and rod photoreceptors). Our data indicated that a significant number of genes required for precursors and mature RGCs were in an inactive (repressive) state (79% and 48% respectively; Fig. 4B, Supplementary Data S6). Most importantly, our data indicated that the key transcription factors for RGC development, *Pou4f2* (Brn3b) and *Isl1*, were in an inactive (repressive) chromatin state (Fig. 5B, Supplementary Data S6). To a lesser degree compared to RGC related genes, a significant number of genes required for precursors and mature bipolar cell differentiation were in an inactive (repressive) state (69% and 53% respectively; Fig. 4B, Supplementary Data S6). The key transcription factor required for bipolar cell development, *Vsx2* (Chx10), was in an inactive (repressive) chromatin state (Fig. 5C, Supplementary Data S6). Yet a significant number of genes required for cone and rod photoreceptor development were in an active/open (permissive) chromatin state. Interestingly, this number is higher in mature photoreceptors (cones: 85%; rods: 100%, Supplementary Data S6) compared to photoreceptor precursors (cones: 81%; rods: 92%) (Figs 4B and 5D, Supplementary Data S6). Our analysis of genes required for cone and rod phototransduction revealed that the majority of these genes are in an active/open (permissive) chromatin state (91% and 96% respectively; Fig. 4B, Supplementary Data S6). Thus, the chromatin state of adult RPE is very close to the photoreceptor epigenetic state; in particular, the state is very close to mature photoreceptors. Meanwhile, the majority of inactive (repressive) states of key genes for RGC, bipolar cell, amacrine, and horizontal cell development are marked by H3K27me3 (chromatin state 5), which is related to the temporarily inactive Polycomb state. These genes may be activated in the presence of some (not yet identified) pioneer transcription factors (PTFs).

### Promoters of photoreceptor and phototransduction-related genes belong to highly methylated regions of RPE genomic DNA

A common feature of genome-scale DNA methylation profiles includes a correlation between DNA methylation and the presence of the H3K9me3 and H3K27me3 markers, corresponding to the permanently inactive (repressive) chromatin state, and the absence of H3K4 methylation^41^. To evaluate the correlation between DNA methylation and histone modification in regulatory regions of retinal cell type genes, and genes required for phototransduction, we used our global epigenetic profile of adult RPE. We expected to observe cases similar to the above (permissive or repressive chromatin states of regulatory regions of retinal cell type and phototransduction genes) dynamics in DNA methylation. Surprisingly, we found the inverse patterns for the tested genes (Fig. 4B, Supplementary Data S6 and S7). Analysis of DNA methylation of promoter regions for all (except photoreceptor) types of retinal neuronal precursors (RGCs, bipolar cells, horizontal cells, amacrine cells) revealed that these regulatory elements were present in unmethylated or low-methylated regions (Fig. 4B, Supplementary Data S7). Similar to precursors, genes required for all retinal neurons (except photoreceptors) were mostly unmethylated or low-methylated (Fig. 4B, Supplementary Data S7). However, we detected a small group of genes in highly-methylated regions (such as *Sncg*, *Dcx*, and *Opn4* for RGCs; *Cabp5* and *Rcvrn* for bipolar cells; *Ddc*, *Slc17a8*, and *Th* for amacrine cells; *Gja10* and *Dcx* for horizontal cells) that may not be critical for development of corresponding phenotypes (Supplementary Data S7). At the same time, our analysis of the RPE methylome revealed that promoters of many genes critical for photoreceptor development were in highly-methylated regions (Fig. 4B, Supplementary Data S7). Our data indicates that promoters of cone photoreceptor genes coding short-wave and middle-wave sensitive opsins (*Opn1sw* and *Opn1mw*), as well as promoters of *Arr3* and *Gngt2*, were highly-methylated (80% and higher; Supplementary Data S7). However, promoters of genes required for rod photoreceptor development were mostly affected. We found that 38% of rod precursor gene promoters and 67% of promoters of genes required for mature rod photoreceptors were highly-methylated. The majority of these genes are critical for rod photoreceptor development. For example, methylation of promoters of *Rho* (coding rhodopsin), *Nrl* and *Nr2e3* (both of them define the rod phenotype), *Rcvrn*, *Pde6a* and *Pde6b* (these genes are critical for phototransduction), and *Mir182* and *Mir96* (regulators of the rod differentiation program) was more than 80% (Supplementary Data S7). Similar to photoreceptor genes, we found that promoters of 41% of genes required for cone phototransduction and 44% of genes required for rod phototransduction were highly-methylated (80% or more). These genes include *Rho* (rod), *Opn1sw* (cone), *Opn1mw* (cone) *Rcvrn* (both), *Arr3* (cone), *Pde6a* (rod), *Pde6b* (rod), *Pde6g* (rod), *Pde6h* (cone), *Grk1* (rod), *Cnga1* (rod), *Gngt1* (rod), *Gngt2* (cone), *Gucy2d* (both), *Gucy2f* (both), and *Rdh8* (both), many of which are critical for phototransduction (Supplementary Data S7). Interestingly, our data indicates that the majority of promoters for these genes contained either the H3K4me1 histone modification or none of the histone marks tested in our study (Supplementary Data S6). These data suggest that phenotypes of cone and rod photoreceptors are silent in RPE because of hypermethylation of promoters for key genes required in photoreceptor development and function. Meanwhile, these genes are not in a repressive chromatin state and their promoters may be accessible for DNA demethylation.

**Figure 6:**
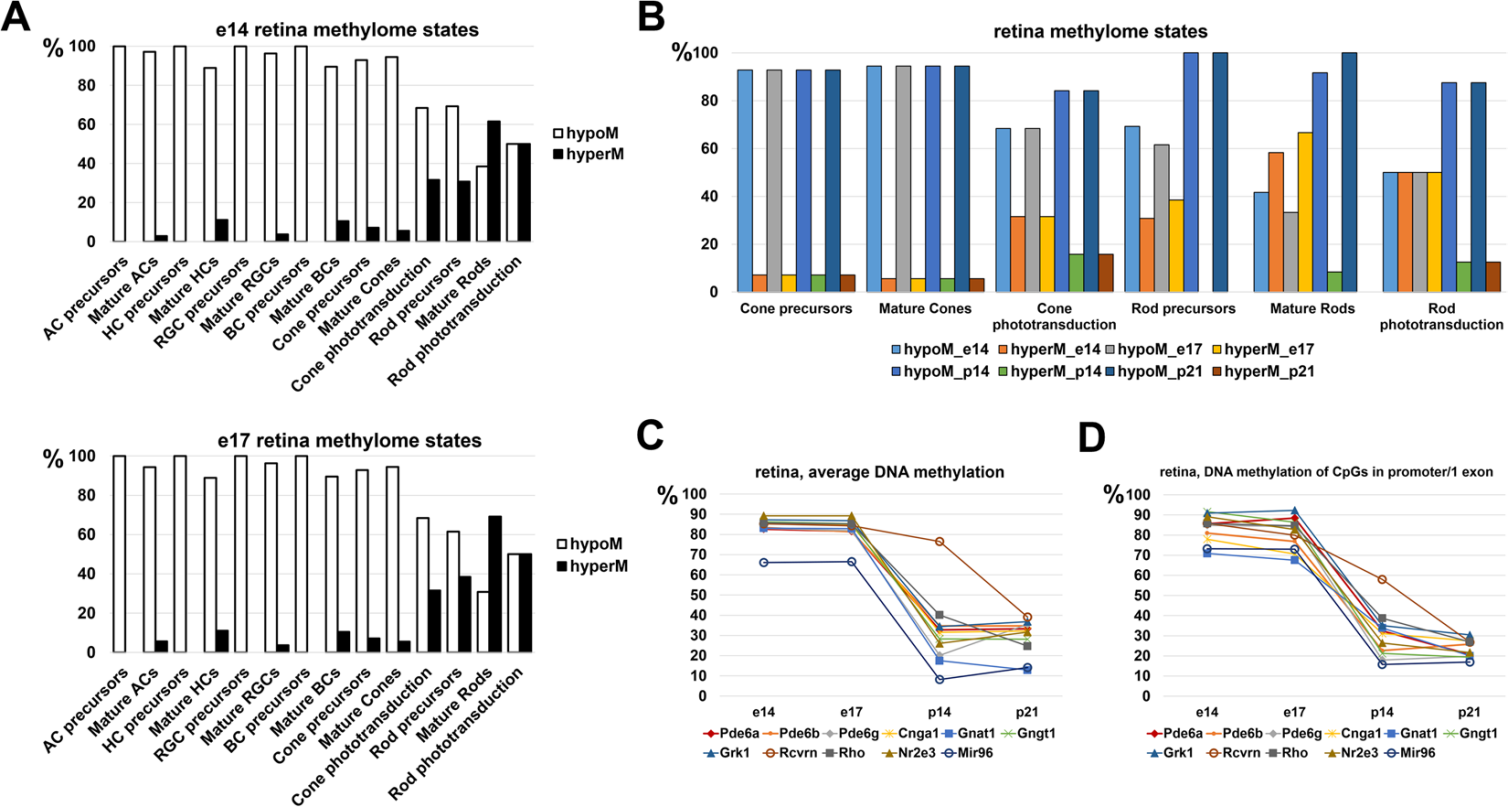
Methylation levels of promoter regions for key genes required in rod photoreceptor development and function are reduced during retinal development (methylation level is high in progenitors and low in photoreceptors). (**A**) The methylome states of E14 and E17 retinas, which contain high numbers of retinal progenitors (especially at E14), indicate low methylation levels in promoters of genes of non-photoreceptor retinal neurons and high methylation levels in rod photoreceptors and phototransduction related genes. (**B**) Methylome states of photoreceptor related genes in the early (E14/E17) and late (P14/P21) stages of retinal development indicate reduced methylation of rod-related genes. (**C**) Average methylation levels in promoter regions of rod and phototranduction related genes were reduced during retinal development. Average methylation level was calculated using the methylKit software package. (**D**) Average methylation levels based on methylation of individual CpGs in the promoter and first exon of rod and phototranduction genes were reduced during retinal development.

Since RPE, retinal progenitor cells (RPCs), and retinal phenotypes are derivatives of the optic vesicle progenitors (OVPs), we asked whether the “methylation of photoreceptor phenotypes” is a part of the mechanism for RPE differentiation. To answer the question, we tested published WGBS data (GEO accession number GSE87064, SubSeries GSE87062)^42^. We used WGBS data from embryonic-day (E) 14 and 17 retinae, which mostly contain RPCs, and WGBS data from postnatal-day (P) 14 and 21 retinae that mostly contain rod photoreceptors. Since the methylome of P14 and P21 retinae reflects the methylome of rod photoreceptors, while WGBS of DNA isolated from E14 and E17 retinae reflects the methylome states of RPCs – which can differentiate into all retinal phenotypes – we first analyzed the methylation levels of key genes required for development of all retinal neurons. We also tested the methylation levels of genes involved in cone and rod phototransduction. Surprisingly, our results were similar to the RPE methylome (Figs 4B and 6A, Supplementary Data S7 and S8). The same RPE methylome genes involved in photoreceptor development and phototransduction were highly-methylated in genomic DNA isolated from E14 and E17 retinas (Supplementary Data S7 and S8). Since photoreceptor phenotypes were most affected, we then tested the DNA methylation dynamics in regulatory regions of photoreceptor and phototransduction related genes during development using WGBS data from E14, E17, P14, and P21 retinae. We found that the level of DNA methylation in promoters of rod-related and rod phototransduction-related genes was reduced during RPC differentiation into rod photoreceptors (Fig. 6B and Supplementary Data S8). Meanwhile, reduced methylation of these gene promoters were correlated with increased expression of these genes (https://pecan.stjude.org/proteinpaint/study/retina2017)^42^. It should be noted that methylation levels of most genes (except *Pde6h*) involved in cone phototransduction were not changed in P14 and P21 retinae. We also identified a list of genes for which methylation was reduced during development (Fig. 6C,D; Supplementary Data S9). Comparison of methylation levels for individual CpGs in promoter regions and the first exons of these genes suggest similar methylation patterns in the adult RPE and embryonic (E14/E17) retinae (mostly containing RPCs; Supplementary Data S9). Our data indicate that during RPC differentiation into photoreceptors, methylation of promoters of photoreceptor-related and phototransduction-related genes is removed. These events may facilitate increased expression of these genes. The methylation of photoreceptor-related genes might not be part of the mechanism of OVP differentiation into RPE.

## Discussion

For the first time, we characterized the global epigenetic profile of adult mammalian RPE cells to understand the epigenetic plasticity of this cell type to differentiate into retinal neurons. Our findings indicate that the majority of RPE promoters are in open (no tested histone marks detected) or active chromatin (permissive chromatin states), which is characteristic of epigenetically mobile stem cells and progenitors^29–31^. This conclusion is supportted by our epigenetic data in which we found a high similarity of RPE chromatin and methylome states with optice vesicle progenitors and RPCs. Our data also supports the observation of RPE’s ability to proliferate and undergo a transition from an epithelial into a mesenchymal state during some retinal diseases (condition known as proliferative vitreoretinopathy - PVR). Our observations indicate that Müller glia related genes are mostly epigenetically available for activation. Meanwhile, analysis of RPE chromatin and methylome states of genes related to retinal neuronal phenotypes revealed inverse epigenetic patterns. Our findings indicate that many key genes required for inhibitory and excitatory (except photoreceptors) retinal neuronal phenotypes are temporarily repressed and may be activated in the presence of corresponding pioneer transcription factors (PTFs). Meanwhile, regulatory elements of key genes required for photoreceptor phenotypes are in a permissive chromatin state, and should be activated since required transcription factors like Otx2, Rorb, Rora, etc. are present in adult RPE. Absence of activation may be due to methylation of the majority of photoreceptor genes. We found that the majority of genes required for photoreceptor phenotypes and for phototransduction is highly-methylated, while genes associated with the rest of retinal neuronal phenotypes are unmethylated or low-methylated in RPE genomic DNA. Our data suggest that RPE and RPCs had very similar methylome patterns, which were eliminated during RPC differentiation into photoreceptors, but stay in adult RPE. Thus, two different mechanisms should be activated to promote retinal neuronal phenotypes in RPE. The first mechanism includes specific PTFs to open repressed chromatin in all precursors of retinal neurons (except photoreceptors). The second mechanism includes demethylation of regulatory elements of photoreceptor related genes. Both mechanisms may be implemented in amphibians to initiate retinal regeneration after injury, but they may be repressed/absent in mammalian RPE.

The eyes begin to develop from the so-called “eye field”, a population of cells in the anterior neural plate that express eye field transcription factors (EFTFs): Rax, Pax6, Six3, Six6, and Lhx2^15–22^. The published literature suggests that broad expression of Otx2 and Sox2 as well as gradient expression of Pax6 and Six3 may promote Rax expression in these cells during eye field formation^15–22^. Subsequently, high levels of Sonic hedgehog (Shh) in the medial neural plate reduces Pax6 expression in the eye field, facilitating its separation into two eye fields, later forming two optic vesicles^15–22^. During optic vesicle formation, EFTFs promote expression of the Lhx2 transcription factor, which is critical for the optic vesicle patterning into RPE, the retina, and the optic stalk^15–22^. Two transcription factors Mitf and Pax2, which respectively regulate RPE and optic stalk development, are initially present throughout the optic vesicle progenitors^15–22^. However, Mitf and Pax2 expression is downregulated by Vsx2 (Chx10) and Pax6, respectively, promoting retinal phenotypes in these progenitors^15–22^. The optic vesicle then invaginates to form the optic cup^15–22^. Lhx2 can activate both Mitf and Vsx2, but activates Mitf first since Vsx2 activation requires close contact of the optic vesicle presumptive retina domain with the surface ectoderm^15–22^. Thus, RPCs and RPE should have close epigenetic memory of optic vesicle progenitors (OVPs). Our data supports this conclusion. We detected the small number of repressed genes required for RPC and OVP phenotypes in RPE. These genes are important for RPCs and OVPs, but the expression of these genes was repressed on the chromatin level by mostly H3K27me3 alone (chromatin state 5) and may be activated in the presence of corresponding PTFs. Importantly, some of these factors such as Sox2 and Ascl1 are themselves PTFs. Our data also indicate that activation of Rax, Six6, and Vsx2 in RPE may be required to promote RPC phenotypes in these cells (Supplementary Data S6). We observed an even bigger list of repressed genes required for amacrine cell, horizontal cell, RGC, and bipolar cell development (Supplementary Data S6) in RPE. Nevertheless, regulatory elements for the majority of these genes are in a temporary inactive (repressive) chromatin state (chromatin state 5) and activity of some PTFs may promote RPC and retinal neuronal phenotypes in RPE. We can speculate that H3K27me3-mediated repression of some of these genes (like Sox2 and Rax) in RPE and activation of other transcription factors (like Vsx2 and Ascl1) by some PTFs in RPCs may regulate differentiation of OVPs into RPE progenitors and RPCs, respectively. Meanwhile, activation of PTFs, like Ascl1, may promote chromatin derepression and activation of retinal neuron phenotypes^23,24^. We can also speculate that these PTFs may be activated in amphibian RPE to initiate retinal regeneration after injury.

While we observed some differences in chromatin states between genes required for OVP, RPC, and RPE phenotypes – and significant differences between RPE chromatin states and all (except photoreceptors) retinal neurons – we did not detect significant differences between genes promoting these cell phenotypes on the DNA methylation level. At the same time, we found that regulatory elements of genes critical for photoreceptor development and function, especially for rod photoreceptors, were highly-methylated. It was at first surprising why the expression of photoreceptor related genes is absent in RPE while Otx2, Rorb, and Rora expression is present in these cells and promoters of key genes for photoreceptor development and function are in an active/open (permissive) chromatin state (Fig. 5D)^15,43^. Our data suggest that high methylation levels of regulatory elements of key photoreceptor genes may prevent expression of these genes in adult RPE. These methylation patterns may be inherited by RPE and RPCs from OVPs. Further, the methylation of photoreceptor and phototransduction related genes stays in RPE, but is eliminated during RPC differentiation into photoreceptors. Emerging evidence suggests that DNA demethylation plays an important role during neurogenesis^44^. The DNA demethylation pathway includes the Ten-Eleven Translocation (TET) proteins, which catalyze the sequential conversion of 5-methylcytosine into 5-hydroxymethylcytosine, then 5-formylcytosine and 5-carboxylcytosine are both converted back to the unmethylated cytosine^45,46^. The TET protein family includes three dioxygenases: Tet1, Tet2, and Tet3^45,46^. Tet1 can regulate adult neurogenesis due to DNA demethylation increasing transcription of target genes^47–50^. Tet2 activation leads to reduced adult neural stem cell proliferation and increased differentiation in adult neurogenesis^51^. Since Tet2 does not have a DNA binding domain, it physically interacts with transcription factors like Foxo3a, which helps to specify target genes that should be activated during adult neurogenesis^51^. The critical role of Tet2 and Tet3 during zebrafish retinal neurogenesis was shown recently^52^. Differentiation of RGCs, and especially photoreceptors, was impaired in zebrafish Tet2/Tet3 double knockouts^52^. Xenopus Tet3 alone can regulate early eye and neural development by directly activating a set of key developmental genes^53^. Meanwhile in mice, Tet3 requires Rest-guided targeting of genes, which should be activated to facilitate photoreceptor differentiation^54^. It should be noted that these studies proposed the important role of 5-hydroxymethylcytosine generated by the TET family as an activator for expression of key genes for neurogenesis and retinogenesis. However, whole-genome bisulfite sequencing cannot separate 5-methylcytosine and 5-hydroxymethylcytosine modifications. Meanwhile, as we noted above, our data suggest reduced methylation in photoreceptor promoters during RPC differentiation into photoreceptors. Thus, TET mediated conversion of 5-methylcytosine into 5-hydroxymethylcytosine is just the first important step in demethylation of photoreceptor-related promoters. The 5-hydroxymethylcytosine modifications may already be able to promote photoreceptor phenotypes, even before the demethylation process is finished. Finally, our data and published literature suggest that methylome dynamics and TET demethylase activity are critical in adult neurogenesis and retinogenesis (especially in photoreceptor differentiation). It should be noted that Tet1-3 expression is very low in RPE (Supplementary Data S1). Therefore, forced activation of TET genes in adult RPE may promote photoreceptor phenotypes in these cells. Since Xenopus Tet3 can regulate eye and neural development, amphibian RPE may use TET proteins to initiate and promote retinal regeneration from adult RPE after injury.

In conclusion, our study of the epigenetic plasticity of adult RPE revealed the similarity of these cells to progenitors with a high ability for EMT. Our findings suggest two mechanisms (inverse epigenetic patterns) that may prevent mammalian RPE from reprogramming and differentiating into retinal neurons: 1) repressive chromatin states prevent amacrine cell, horizontal cell, RGC, and bipolar cell phenotypes; 2) high methylation levels abolish cone and mostly rod photoreceptor phenotypes. PTF activity and DNA demethylation pathways in amphibian RPE may facilitate retinal regeneration in these animals after injury. Such activity may have been lost in mammalian RPE, or perhaps amphibian RPE may have obtained it during evolution. Our results suggest that certain PTFs, which remain unidentified, and forced TET demethylase expression may be required to restore retinal regeneration abilities after injury in adult mammalian RPE.

## Methods

### Animals

All experiments were performed in compliance with the National Institutes of Health (NIH) Guide for the Care and Use of Laboratory Animals, the Association for Research in Vision and Ophthalmology (ARVO) statement for use of animals in ophthalmic and vision research, and the University of Miami Institutional Animal Care and Use Committee’s (IACUC) approved protocol. C57BL/6 J (stock number 000664) mice and B6.Cg-Tg(Nrl-EGFP)1Asw/J (stock number 021232) were obtained from the Jackson Laboratory (Bar Harbor, Maine, United States). Mice were housed under standard conditions of temperature and humidity, with a 12-hour light to dark cycle and free access to food and water.

### Isolation of adult RPE cells

Eyes from 10 week old mice were enucleated following CO_2_-induced euthanasia. RPE cells were harvested by trypsin digestion, as described previously^26^. Briefly, after the lens was removed, the eyes were incubated in hyaluronidase (1 mg/mL) (Sigma Aldrich, St Louis, MO cat. no. H-3506) for 45 min in the HBSS-H- (HBSS without calcium, without magnesium buffer + 10 mM HEPES) buffer at 37 °C. The eyes were then kept for 30 min in HBSS-H + (HBSS containing Ca^2+^ and Mg^2+^ and 10 mM HEPES) buffer at 4 °C. After incubation, the cornea, iris epithelium, and retina were removed and the eyecups were incubated in 0.05% trypsin-EDTA (Life Technologies, Grand Island, NY) for 45 min at 37 °C. To inactivate trypsin, the eyecups were kept in 20% fetal bovine serum (FBS) (Hyclone, GE Healthcare, Pittsburgh, PA) in HBSS-H + buffer. To detach RPE sheets, the eyecups were then shaken. Finally, RPE sheets were centrifuged at 340 × g for 2 min and the pellet was used for: (1) RNA purification (qRT-PCR, microarray study); (2) DNA purification (whole genome bisulfite sequencing); (3) ChIP-seq study.

### Quantitative RT-PCR analysis

Quantitative RT-PCR analysis was performed as described previously using gene-specific primers (Supplementary Data S10)^55,56^. Briefly, RNA samples were extracted from adult RPE cells using the Absolutely RNA® Nanoprep kit (Agilent Technologies, Santa Clara, CA) and reverse transcribed with SuperScript III Reverse Transcriptase (ThermoFisher Scientific, Grand Island, NY) to synthesize cDNA. Quantitative PCR was then performed (Rotor-Gene Q, Qiagen, Valencia, CA) using a kit (SYBR GREEN PCR MasterMix; Qiagen, Valencia, CA). Relative expression was calculated by comparison with a standard curve following normalization to expression of the housekeeping gene *Gapdh*, chosen as a control. Data are presented as a fold-change of the corresponding value for *Gapdh* ± SEM. Quantitative RT-PCR measurements were analyzed with the Student’s t-test. Values of P < 0.05 were designated as statistically significant.

### Immunohistochemistry

RPE sheets were fixed in 4% PFA and blocked with 5% normal donkey serum with 0.15% Tween-20 in PBS at pH 7.4. Cells were then incubated with the primary antibody (Supplementary Data S10) followed by species-specific secondary fluorescent antibodies (Invitrogen, Carlsbad, CA). Negative controls were incubated with the secondary antibody only. Imaging was performed with a confocal laser microscope (Leica TSL AOBS SP5; Leica Microsystems).

### RNA extraction, probe preparation, and array hybridization

RNA samples were extracted from adult RPE cells using the Absolutely RNA® Nanoprep kit (Agilent Technologies, Santa Clara, CA). RNA samples were sent to Ocean Ridge Biosciences (Palm Beach Gardens, FL, USA) for analysis using mouse exonic evidence-based oligonucleotide (MEEBO) microarrays. Biotin-labeled complementary RNA was made from total RNA according to Van Gelder’s protocol^57^. Biotinylated complementary RNA samples were fragmented, diluted in a formamide-containing hybridization buffer, and loaded on to the MEEBO microarray slides enclosed in custom hybridization chambers (for more information on the MEEBO oligonucleotide set please refer to http://alizadehlab.stanford.edu/). The slides were hybridized for 16–18 hours in a Model 400 hybridization oven (Scigene, Sunnyvale, CA). After hybridization, the microarray slides were washed under stringent conditions, stained with Streptavidin-Alexa-647 (Invitrogen, Carlsbad, CA), and scanned using an Axon GenePix 4000B scanner (Molecular Devices, Sunnyvale, CA).

### Microarray data analysis

Spot intensities for each probe were calculated by subtracting median local background from median local foreground for each spot. The spot intensities were then normalized. After removing data for low quality spots, the mouse probes’ intensities were filtered to identify all probes with an intensity above a normalized threshold (untransformed normalized signal intensity >500). The mean signal intensity and standard deviation (SD) can be found in Supplementary Data S1.

### ChIP-Seq

Since some photoreceptor related genes belong to the X chromosome, we used RPE sheets isolated from male mice only, to avoid exclusion of this chromosome from analysis in this experiment. All antibodies were validated by Diagenode Inc. Freshly isolated RPE were cross-linked for 9 min at room temperature in 1% formaldehyde (Sigma-Aldrich, F8775-25ML) in 1X PBS. To stop the cross linking reaction, glycine (Sigma-Aldrich, G-7403) was added to a final concentration of 0.125 M. From this point onwards, we worked on ice. The cells were centrifuged at 300 × g for 10 minutes at 4 °C and the supernatant was aspirated. The cross-linked cells were washed in 1 ml of ice cold HBSS containing a protease inhibitor cocktail (PIC, 200x; final concentration 1x; Sigma-Aldrich, P8340). The cells were centrifuged again at 300 × g for 10 minutes at 4 °C, the supernatant was discarded, and the cell pellets were stored at −80 °C. The ChIP-seq experiment was conducted by Diagenode ChIP-seq profiling service. The chromatin was prepared using the True MicroChIP Kit (Diagenode Cat# C01010130). Chromatin was sheared using the Bioruptor® Pico sonication device (Diagenode Cat# B01060001) combined with the Bioruptor® Water cooler for 7 cycles using a 30” [ON] 30” [OFF] settings. Shearing was performed in 0.65 mL Bioruptor® Pico Microtubes (Diagenode Cat# C30010011) with the following cell number: 14,000–28,000 in 100 μL. 50 μL of this chromatin was used to assess the size of the DNA fragments obtained by High Sensitivity NGS Fragment Analysis Kit (DNF-474) on a Fragment Analyzer™ (Advanced Analytical Technologies, Inc.). ChIP was performed using IP-Star® Compact Automated System (Diagenode Cat# B03000002) following the protocol of the aforementioned kit. Chromatin corresponding to 14,000–28,000 cells was immunoprecipitated using the following antibodies and amounts: H3K4me1 (0.5 µg; Diagenode Cat# C15410194), H3K4me3 (0.5 µg; Diagenode Cat# C15410003–50), H3K9me3 (0.5 µg; Diagenode Cat# C15410193) and H3K27me3 (0.5 µg; Diagenode Cat# C15410195). Chromatin corresponding to 10% was set apart as Input. The DNA after reverse cross-linking is quantified using Qubit™ dsDNA HS Assay Kit (Thermo Fisher Scientific, Q32854). Moreover, qPCR analysis was made to check ChIP efficiency using the following primers *Gapdh*, *Prm1*, and *Gas2l1* (Additional file 19). Libraries were prepared from the input and ChIP’d DNA (500 pg) using MicroPlex Library Preparation Kit v2 (12 indices) (Diagenode Cat# C05010013). Library amplification is assessed using High Sensitivity NGS Fragment Analysis Kit (DNF-474) on a Fragment Analyzer™ (Advanced Analytical Technologies, Inc.). Libraries were then purified using Agencourt® AMPure® XP (Beckman Coulter) and quantified using Qubit™ dsDNA HS Assay Kit (Thermo Fisher Scientific, Q32854). Finally their fragment sizes were analyzed using the High Sensitivity NGS Fragment Analysis Kit (DNF-474) on a Fragment Analyzer™ (Advanced Analytical Technologies, Inc.). When the proportion of fragments >500 bp was too high, libraries were subjected to a double size selection using Agencourt® AMPure® XP (Beckman Coulter).

### ChIP-Seq data analysis

Libraries were pooled and sequenced on an Illumina HiSeq 4000 with single-end reads of 50 bp length, running HiSeq Control Software HD version 3.4.0.38. Quality control of sequencing reads was performed using FastQC (http://www.bioinformatics.babraham.ac.uk/projects/fastqc). Reads were then aligned to the reference genome (mm10) obtained from the UCSC genome browser using BWA software v.0.7.5a^58^. Samples were filtered for regions blacklisted by the ENCODE project. Subsequently, samples were deduplicated using SAMtools version 1.3.1^59^. Alignment coordinates were converted to BED format using BEDTools v.2.17 and peak calling was performed using SICER with customized parameters for each histone mark^60^. To integrate our ChIP-seq data and identify the major combinatorial and spatial patterns of marks (so-called chromatin states), we used ChromHMM software (http://compbio.mit.edu/ChromHMM/) according to the manual^27^. Annotation of identified peaks and segments was carried out with the R Bioconductor package Annotatr^37^.

### Whole genome bisulfite sequencing (WGBS)

Since some photoreceptor related genes belong to the X chromosome, we used RPE sheets isolated only from male mice for the methylation analysis to avoid exclusion of this chromosome from the study. Genomic DNA was purified from RPE samples using the DNeasy Blood & Tissue Kit (QIAGEN #69504). DNA concentration of the samples was measured using the Qubit® dsDNA BR Assay Kit (Thermo Fisher Scientific). DNA quality of the samples was assessed with the Fragment AnalyzerTM and the DNF-487 Standard Sensitivity genomic DNA Analysis Kit (Advanced Analytical). WGBS was conducted by the Diagenode Inc. Genomic DNA was sheared using the Bioruptor® Pico sonication device (Diagenode Cat# B01060001) combined with the Bioruptor® Water cooler for 15 cycles using a 30” [ON] 30” [OFF] settings. Shearing was performed in 0.2 mL Bioruptor® Pico Microtubes with Caps (Diagenode Cat# C30010020). 1 μL of this sample was used to assess the size of the DNA fragments obtained by a High Sensitivity DNA chip for the 2100 Bioanalyzer (Agilent). DNA concentration of the sample was measured after shearing using the Qubit® dsDNA BR Assay Kit (Thermo Fisher Scientific). WGBS libraries were prepared using the Whole Genome Bisulfite Sequencing (RRBS) Kit (Diagenode Cat# C02030034) following the kit manual. 1 μg of sheared genomic DNA were used to start library preparation for each sample. Following library preparation, samples were bisulfite converted and amplified by PCR using 9 amplification cycles. Final PCR clean-up was performed twice using a 1.1x beads:sample ratio of Agencourt® AMPure® XP (Beckman Coulter). DNA concentration of the libraries was measured using the Qubit® dsDNA HS Assay Kit (Thermo Fisher Scientific). The library profiles were checked using the High Sensitivity DNA chip for the 2100 Bioanalyzer (Agilent). WGBS libraries were sequenced on a HiSeq3000 (Illumina) using 150 bp Paired-end sequencing (PE150).

### WGBS data analysis

The sequenced reads were controlled for quality of sequencing with FastQC tool. Adapter removal was performed using Trim Galore! version 0.4.5 (https://www.bioinformatics.babraham.ac.uk/projects/trim_galore/). The cleaned reads were then aligned to the Mus musculus reference genome (Genome Reference Consortium 37, mm10) using bismark v0.16.1^61^. The average read coverage for Exp1_RPE and Exp1_RPE was 11. The cytosine2coverage and bismark_methylation_extractor modules of bismark were used to infer the methylation state of all cytosines in CpG, CHH, or CHG contexts (for every single mappable read) and to compute the percentage of methylation for each CpG site. DNA methylation analysis from high-throughput bisulfite sequencing results were performed using Bioconductor R packages “MethylSeekR” and “methylKit” according to software documentation^32,35^. Annotation of identified segments and regions was carried out with the R Bioconductor package Annotatr.

## Supplementary information

Supplementary Data S1

Supplementary Data S2

Supplementary Data S3

Supplementary Data S4

Supplementary Data S5

Supplementary Data S6

Supplementary Data S7

Supplementary Data S8

Supplementary Data S9

Supplementary Data S10

## Data Availability

The raw files from the microarray study, ChIP-Seq, and WGBS have been deposited in the NCBI Gene Expression Omnibus (GEO) database. They are accessible through GEO accession number GSE120592.
