# Supplementary material for: The epigenetic basis for the impaired ability of adult murine retinal pigment epithelium cells to regenerate retinal tissue
Source: Sci Rep. 2019 Mar 7;9:3860. doi: 10.1038/s41598-019-40262-w (PMC6405859; doi:10.1038/s41598-019-40262-w)

Title: The epigenetic basis for the impaired ability of adult murine retinal pigment epithelium cells to regenerate retinal tissue

Galina Dvoriantchikova, Bascom Palmer Eye Institute, Department of Ophthalmology, University of Miami Miller School of Medicine, Miami, FL, 33136, USA

Rajeev J. Seemungal, Bascom Palmer Eye Institute, Department of Ophthalmology, University of Miami Miller School of Medicine, Miami, FL, 33136, USA

Dmitry Ivanov *, Bascom Palmer Eye Institute, Department of Ophthalmology, University of Miami Miller School of Medicine, Miami, FL, 33136, USA; Department of Microbiology and Immunology, University of Miami Miller School of Medicine, Miami, FL, 33136, USA; divanov@med.miami.edu.

Supplementary Data S5: Evidences that the RPE methylome does not have partially methylated domains (PMDs)

A

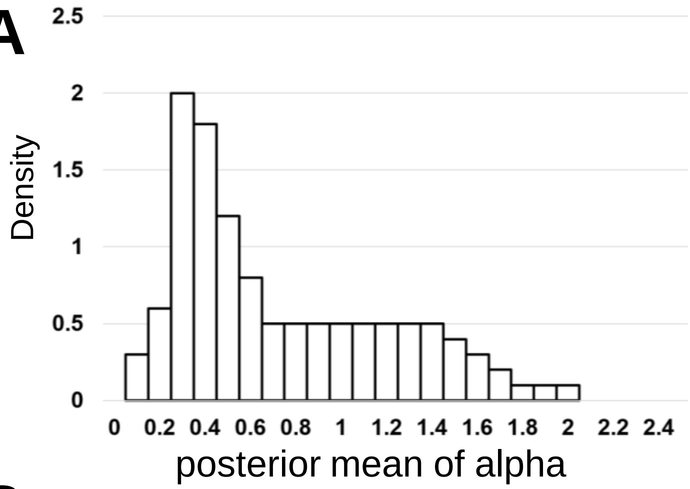

B

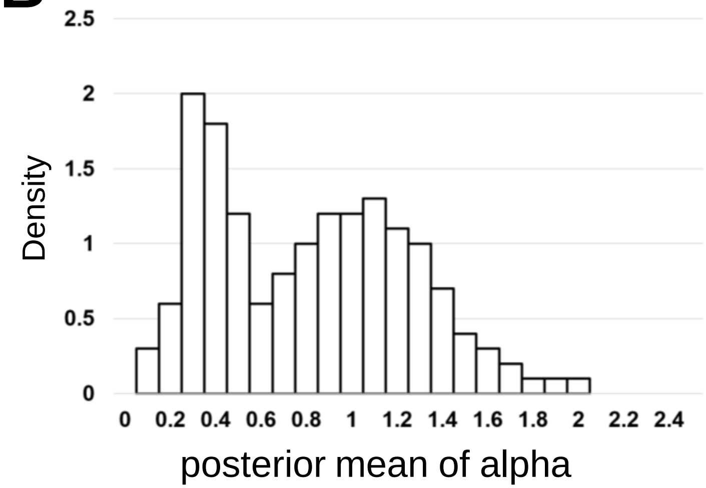

These partially methylated domains (PMDs) are present in the genome if the distribution of α-values is long-tailed (the main peak and the tail) with a significant fraction of α values larger than or equal to 1 (A) or bimodal (the two peaks; B).

The α-value is the distribution of methylation levels in sliding windows comprising 100 consecutive CpGs alongside the genome (Burger et al., Nucleic Acids Res, Jul 2013).

α-values less than 1 define a polarized distribution, indicating the presence of low- and high-methylation regions in the genome.

α-values ≥ 1 are distributions that are rather uniform or reflect intermediate methylation levels (as in PMDs).

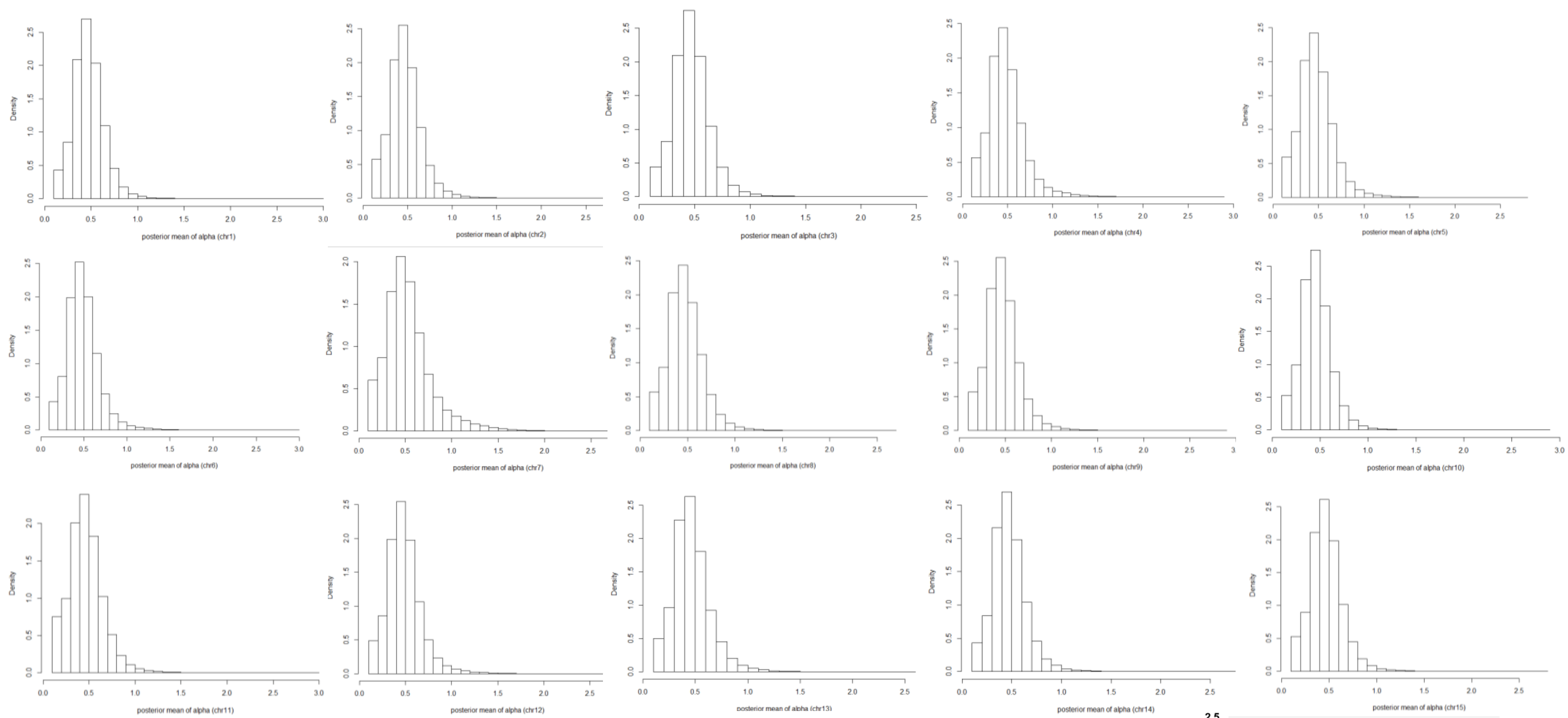

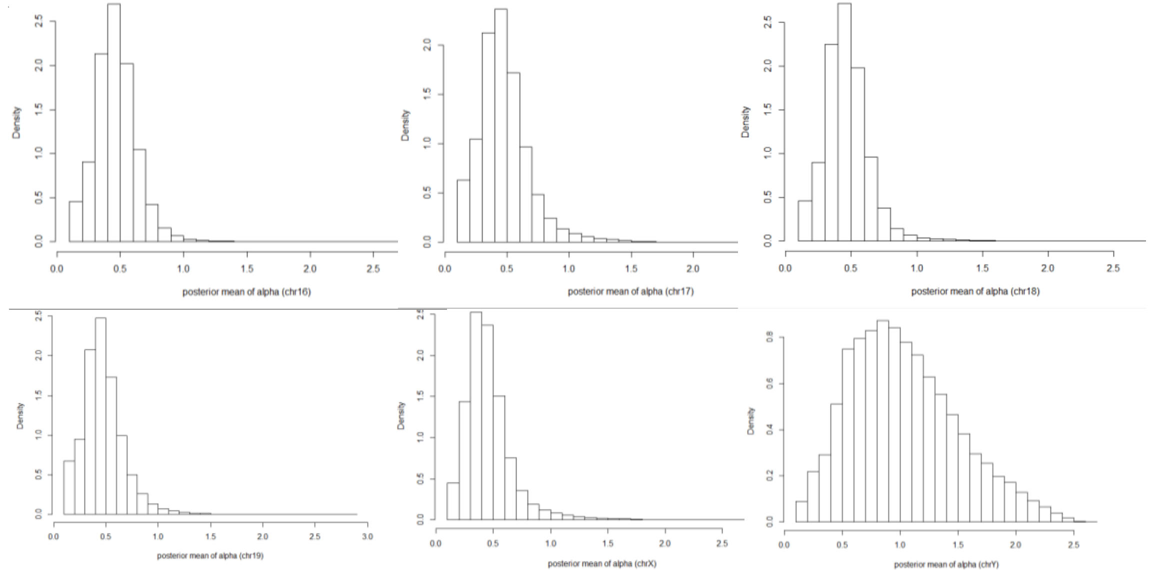

Exp1_RPE
compare to

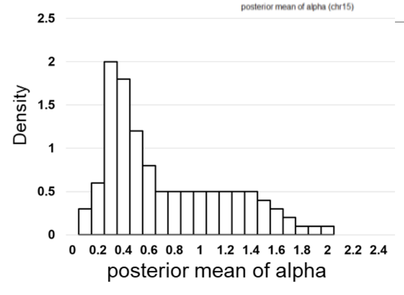

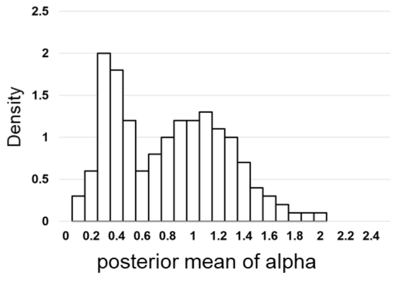

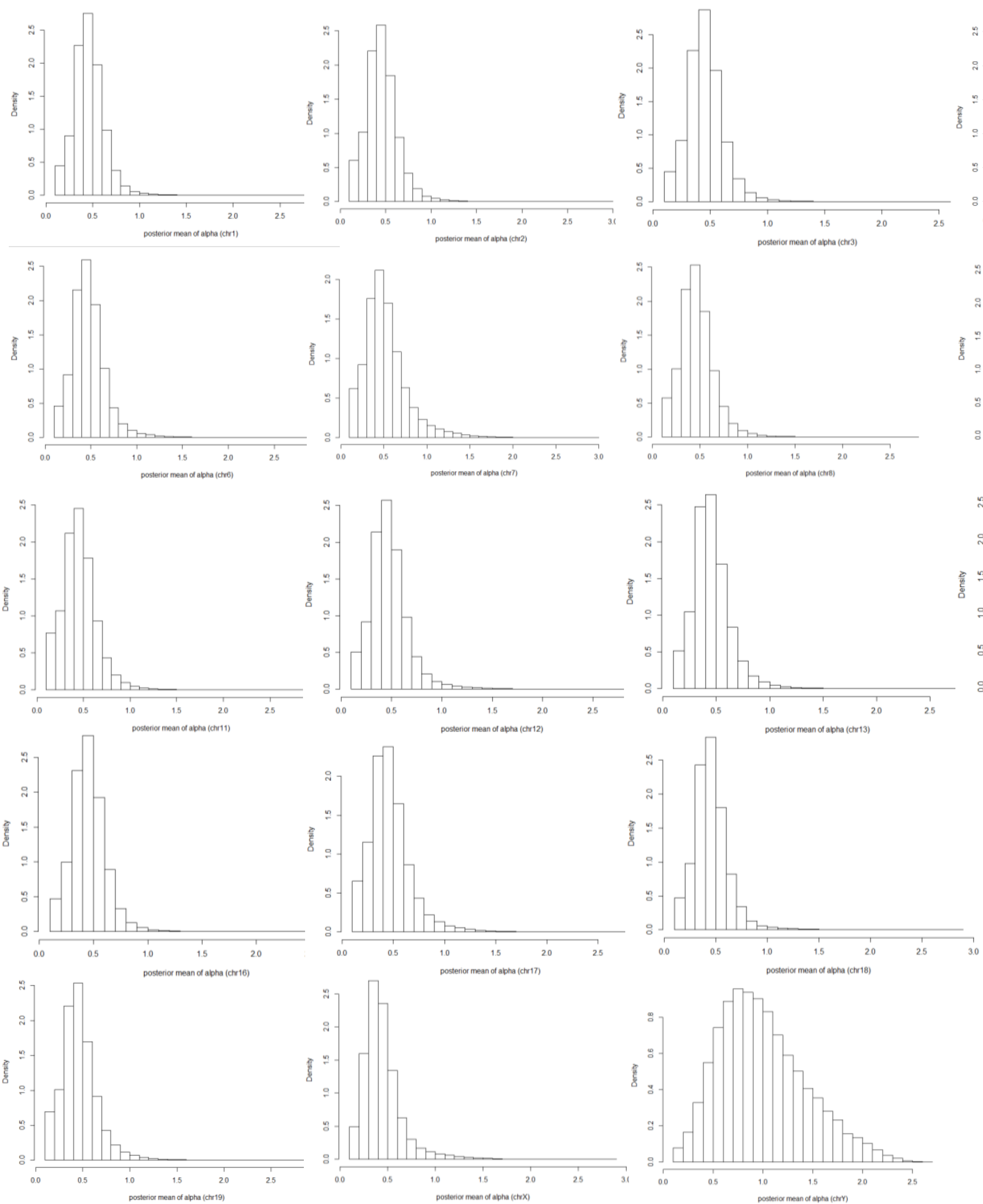

Exp2_RPE compare to

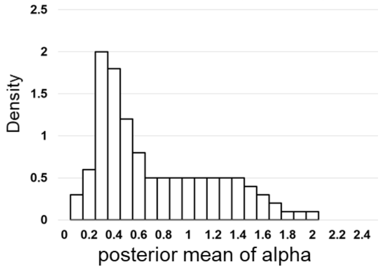

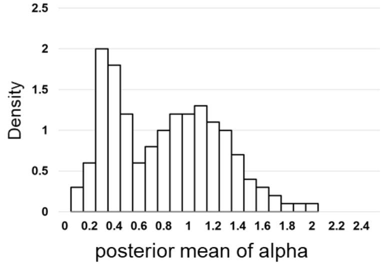

Supplement: Supplementary file 5 — Supplementary Data S5 [file 41598_2019_40262_MOESM5_ESM.pdf]
